# Antenatal Diagnosis of Complete Annular Pancreas With Postnatal Surgical Follow-Up: A Case Report

**DOI:** 10.7759/cureus.67837

**Published:** 2024-08-26

**Authors:** Akshita Panpalia, Suresh Phatak, Prashant Onkar, Kajal Mitra, Wajid Attar

**Affiliations:** 1 Radiodiagnosis, N. K. P. Salve Institute of Medical Sciences and Research Centre and Lata Mangeshkar Hospital, Nagpur, IND; 2 Radiodiagnosis, Datta Meghe Institute of Medical Sciences, Wardha, IND

**Keywords:** antenatal anomaly, intestinal obstruction, pancreatic anomaly, duodenal atresia, annular pancreas

## Abstract

Annular pancreas is a rare congenital disorder, which is characterized by partial or complete pancreatic tissue surrounding the second part of the duodenum. It can be diagnosed antenatally on ultrasound. We are reporting a case where a double bubble sign on an antenatal scan was seen, which was followed up after the delivery of the baby, and radiography also confirmed the findings. Both investigations suggested duodenal obstruction (duodenal atresia and annular pancreas were kept as possible causes). The child was taken for surgery and pancreatic tissue surrounding the second part of the duodenum was seen, confirming the diagnosis of annular pancreas.

## Introduction

Annular pancreas is one of the rare congenital malformations that is characterized by the formation of a band or ring of cephalic pancreatic tissue completely or partially encircling the duodenum in its second part. The pancreatic tissue encases the D2 segment of the duodenum usually close to the ampulla of Vater [1]. The clinical presentation is mostly characterized by causing duodenal obstruction in early life. Few of these can also be diagnosed antenatally [2].

## Case presentation

**Figure 1:**
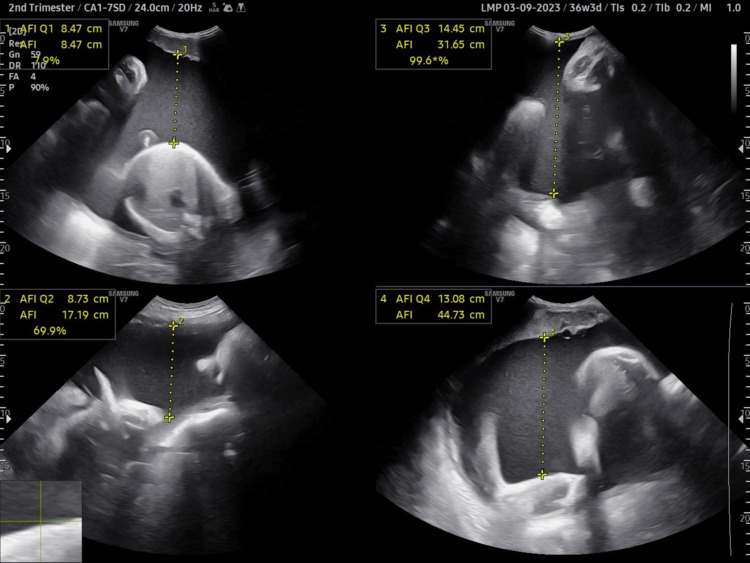
On antenatal ultrasound, the deepest vertical pool measures 14.45 cm, and the amniotic fluid index (AFI) measures 44.73 cm (more than 95th percentile for the corresponding gestational age) suggestive of polyhydramnios.

**Figure 2:**
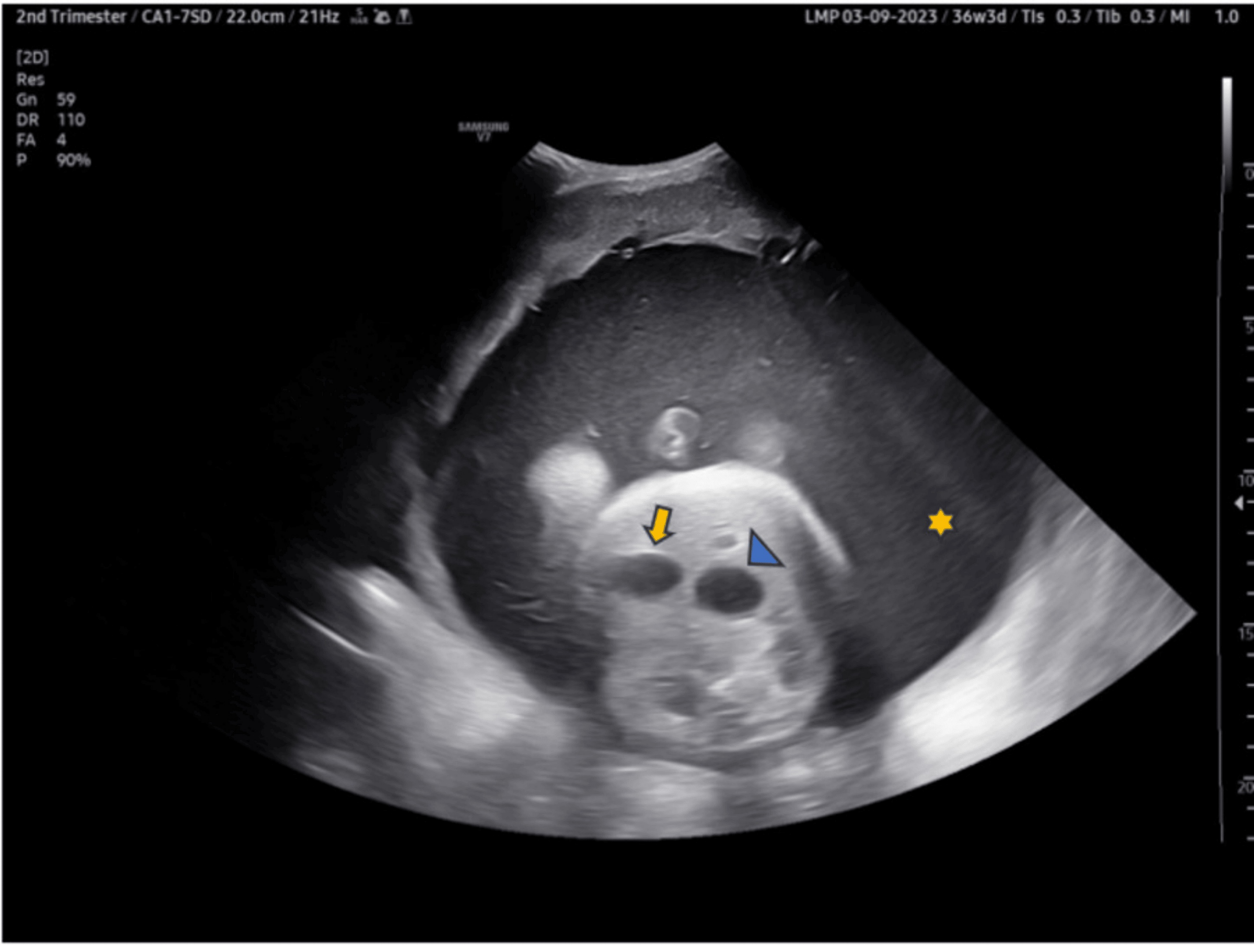
Antenatal ultrasound showing increased liquor (polyhydramnios, blue star) with two cystic structures in the upper abdomen of the fetus suggestive of a double bubble sign and representing a distended fetal stomach (blue arrowhead) and proximal duodenum (yellow arrow).

A 30-year-old primigravida, with a history of severe abdominal distension and respiratory distress, came for a routine antenatal ultrasound at 36 weeks and six days of gestational age. She had undergone only one antenatal care (ANC) scan before, done at 10 weeks, which was normal. The present ultrasound scan showed severe polyhydramnios, with an amniotic fluid index of 44.7 cm (single deepest vertical pocket measuring 14.55 cm) (Figure 1). However, the fetal growth and Doppler parameters were normal. In the fetal abdomen, two dilated structures were seen - the stomach and duodenum, giving a double bubble appearance. However, no echogenic band between these two bubbles was observed (Figure 2). No other abnormality was detected. The diagnosis of duodenal obstruction was given. Differential diagnosis includes duodenal atresia and annular pancreas.

**Figure 3:**
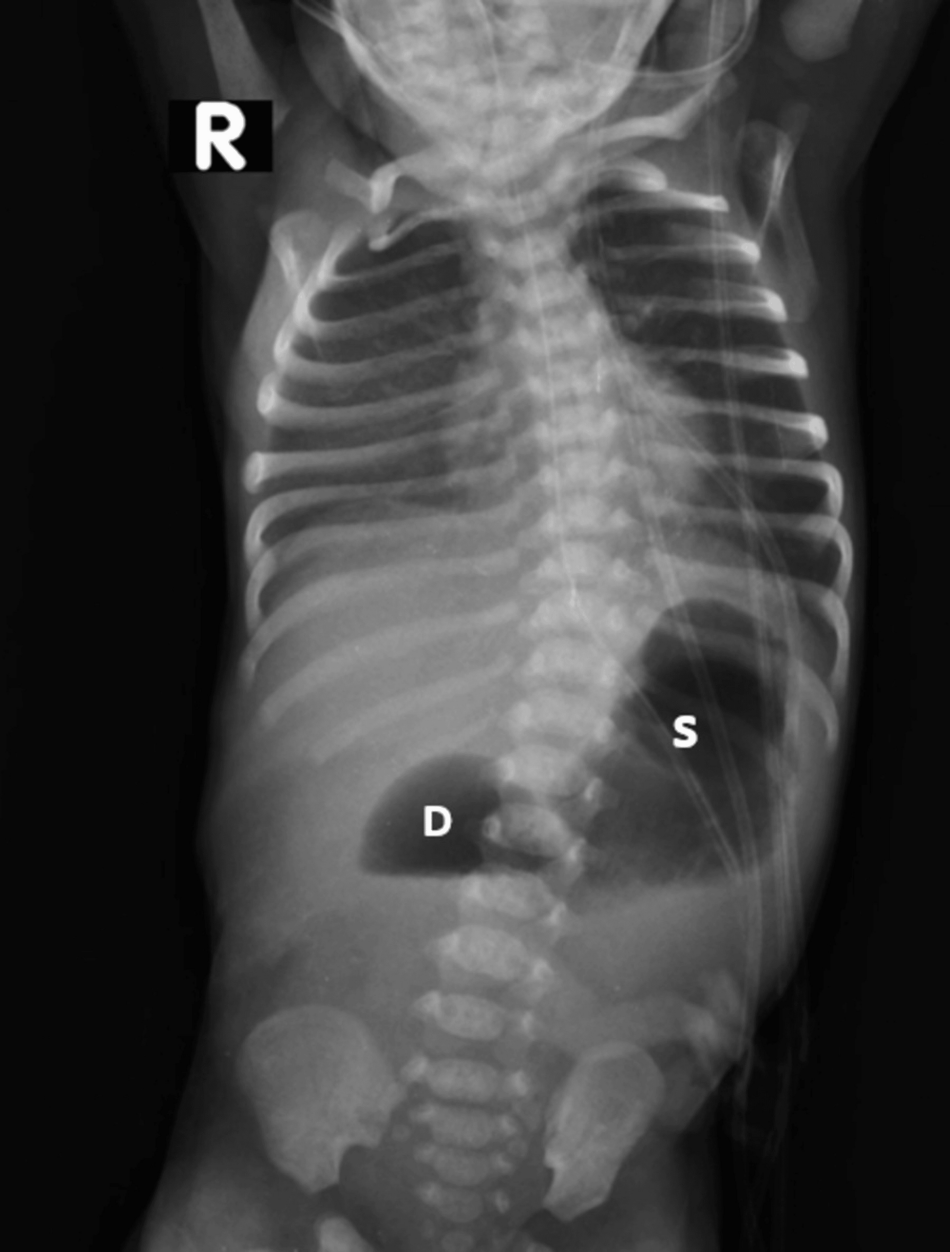
Supine radiograph of the abdomen demonstrates a dilated stomach (denoted by S) and an accompanying dilated proximal duodenum (denoted by D). There is no gas in the bowel distal to the dilated duodenum. This is called the "double bubble" sign.

Due to maternal respiratory distress, elective surgery was planned, and the baby was delivered at 37 weeks. The delivery went uneventful. A 2.8 kg baby boy was delivered. Radiography and sonography were done immediately in the postnatal period, which was also consistent with the antenatal diagnosis. Conventional radiography demonstrated a double bubble sign - a first distended bubble of the air-filled stomach (S) and another of proximal duodenum (D) (Figure 3). As annular pancreas can rarely lead to complete duodenal obstruction and due to the inability to visualize the hyperechoic band around the duodenum, the provisional diagnosis of duodenal atresia was more likely as the cause. No other anomaly was seen in the baby after a thorough clinical examination.

**Figure 4:**
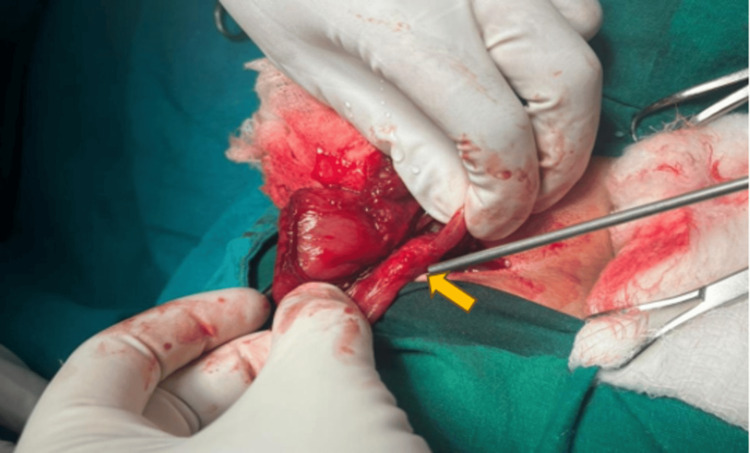
Intraoperative photograph showing a tissue (yellow arrow) covering the second part of the duodenum (part of bowel held by the surgeon).

A laparotomy was performed, and a tissue covering the second part of the duodenum was found, confirming the diagnosis of a complete annular pancreas (Figure 4). Gastro-duodenal anastomosis was performed. The baby was discharged following an uneventful post-operative period.

## Discussion

Usually, the asymptomatic cases of annular pancreas go undiagnosed as no antenatal or postnatal screening standards are established. Hence, the overall incidence is not clear [2]. During the fifth to seventh week of gestation, the pancreas develops from dorsal and ventral pancreatic buds. Normally, the two pancreatic buds rotate along with the rotation of the intestine. When the duodenum rotates clockwise, the ventral pancreatic bud migrates inferiorly and posteriorly and merges with the pancreatic head forming the lower portion of the head of the pancreas and the uncinate process. The dorsal bud forms the rest of the pancreas. This rarely diagnosed congenital disorder is due to ventral bud malrotation, resulting in encasement of the duodenum [3].

Annular pancreas can be partial or complete. Complete annular pancreas usually presents in the antenatal or neonatal period with duodenal obstruction. However, partial ones can present later in life with complications or can even go undiagnosed throughout life [4].

Its association with other congenital abnormalities has also been seen. Significant cardiac anomalies can include atrioventricular canal, ventricular septal defect, cleft mitral valve, atrial septal defect, and tetralogy of Fallot [5]. Situs inversus, tracheoesophageal fistula, nonrotation, phalocele, intestinal malrotation, duodenal atresia, and mobile right colon were also observed in some cases. Down syndrome is the most common chromosomal anomaly associated with it [6].

Radiological investigations are the gold standard investigation for the diagnosis of annular pancreas. Ultrasound not only helps in investigating neonates and children with duodenal obstruction but also suspects it antenatally. However, duodenal obstruction is not only suggestive of annular pancreas. It only suggests that there is a narrowing of the duodenum, which may be caused by many other pathologies such as duodenal atresia or stenosis [2]. 

Duodenal obstruction diagnosis is established based on the “double bubble“ image in a transverse scan of the abdominal cavity, which is in continuation with each other. This typical appearance can be observed on the axial section of the fetal abdomen. This appearance results from a distended stomach on the left side of the abdomen and a distended duodenal bulb in the midline of the abdomen, slightly towards the right. The sign is typically observed in the second trimester. Apart from this, the criteria for diagnosis of annular pancreas include pancreatic tissue identified encircling the second part of the duodenum [2]. It is also important to demonstrate the expansion of the stenotic segment during a peristaltic wave because the peristaltic wave sometimes causes temporary narrowing of the duodenum, giving the appearance of obstruction. Other rare causes of visualization of two bubbles in the abdomen should also be excluded such as splenic cyst, hepatic cyst, choledochal cyst, renal cyst, omental cyst, ovarian cyst, or stomach duplication [7].

Ultrasound and plain abdominal X-ray are the primary radiological investigations used to diagnose pediatric patients. The neonates present with an inability to feed and other signs of duodenal obstruction, and few patients present with pancreatitis. In infants, post-feed vomiting with loss of weight, hypotonia, or signs of dehydration can also be seen. On ultrasound, two distended parts of the stomach and duodenum can be demonstrated. Sometimes, an echogenic structure is seen surrounding the duodenum. On abdominal radiography, two gas-filled structures are seen giving the typical double bubble appearance. Barium studies can also be performed. It shows that the stomach is enlarged, and the enlarged D2 segment of the duodenum is coated with barium [8].

Ultrasound is the preferred modality over others as it is dynamic and, unlike CT/MRI, does not require sedation and is less time-consuming. Other investigations that can also help in the diagnosis are MR cholangiopancreatography, endoscopic retrograde cholangiopancreatography (ERCP), and endoscopic ultrasonography. However, the final diagnosis can only be established through surgery [1].

The definitive surgical procedure includes bypassing the obstructed part of the duodenum as the pancreas cannot be removed completely, which can cause leakage and other complications [9].

## Conclusions

Annular pancreas being a less common congenital pathology and affecting a small number of population, its diagnosis in the antenatal period requires expertise and a high degree of suspicion, along with knowledge of other causes of duodenal obstruction. This case highlights the significance of both antenatal and postnatal diagnosis in managing such cases. Early detection during pregnancy enabled timely planning and intervention postnatally, ensuring prompt surgical correction.
